# How cells align to structured collagen fibrils: a hybrid cellular Potts and molecular dynamics model with dynamic mechanosensitive focal adhesions

**DOI:** 10.3389/fcell.2024.1462277

**Published:** 2025-01-06

**Authors:** Koen A. E. Keijzer, Erika Tsingos, Roeland M. H. Merks

**Affiliations:** ^1^ Mathematical Institute, Faculty of Science, Leiden University, Leiden, Netherlands; ^2^ Institute of Biology Leiden, Faculty of Science, Leiden University, Leiden, Netherlands

**Keywords:** anisotropic extracellular matrix, focal adhesions, cellular Potts model, cell spreading, cell morphology, mechanical reciprocity, mathematical biology

## Abstract

Many mammalian cells, including endothelial cells and fibroblasts, align and elongate along the orientation of extracellular matrix (ECM) fibers in a gel when cultured *in vitro*. During cell elongation, clusters of focal adhesions (FAs) form near the poles of the elongating cells. FAs are mechanosensitive clusters of adhesions that grow under mechanical tension exerted by the cells’ pulling on the ECM and shrink when the tension is released. In this study, we use mathematical modeling to study the hypothesis that mechanical reciprocity between cells and the ECM is sufficient for directing cell shape changes and orientation. We show that FAs are preferentially stabilized along the orientation of ECM fibers, where cells can generate higher tension than in directions perpendicular to the ECM fibers. We present a hybrid computational model coupling three mathematical approaches: first, the cellular Potts model (CPM) describes an individual contractile cell; second, molecular dynamics (MD) represent the ECM as a network of cross-linked, deformable fibers; third, a set of ordinary differential equations (ODEs) describes the dynamics of the cell’s FAs, in terms of a balance between assembly and a mechanoresponsive disassembly. The resulting computational model shows that mechanical reciprocity suffices for stiffness-dependent cell spreading, local ECM remodeling, and ECM-alignment-dependent cell elongation. These combined effects are sufficient to explain how cell morphology is influenced by the local ECM structure and mechanics.

## 1 Introduction

The extracellular matrix (ECM) plays a crucial role in development and in disease. For example, the ECM plays a role in cancer cell migration (Najafi et al., 2019; Yamaguchi et al., 2005), wound healing (Maquart and Monboisse, 2014; Diller and Tabor, 2022), and angiogenesis (Stupack and Cheresh, 2002). The ECM is a complex collection of large fibers such as collagen, fibronectin, and other proteins (Theocharis et al., 2016). The orientation of fibers in the ECM plays an important role in tumor vascularization (Balcioglu et al., 2016), mechanical cell–cell communication (Nahum et al., 2023), and blood clot formation (Kim O. V. et al., 2017). The ECM is continuously remodeled by cells both chemically, through the synthesis and degradation of ECM fibers and associated components, and mechanically, by pulling and reorienting fibers (Theocharis et al., 2016; Winkler et al., 2020). As ECM remodeling leads to local changes in ECM properties such as stiffness, structure, density, and isotropy, to which cells respond through changes in adhesion, cell contraction, or pseudopod extension (Reinhart-King et al., 2005; Malandrino et al., 2019; Doyle et al., 2022), there is a bidirectional chemical and mechanical reciprocity between the cells and the ECM. In this work, we focus specifically on the mathematical modeling of mechanical cell–ECM reciprocity in fibrous ECM, in particular the role of ECM isotropy. For mathematical models of other forms of cell–ECM reciprocity, we refer to Daub and Merks (2013); van Oers et al. (2014); Rens and Merks (2017), Rens and Merks (2020); Chiang and Chung (2024).

The present study attempts to provide mechanistic explanations for three behaviors of cells on fibrous matrices: (1) cell spreading as a function of ECM stiffness, (2) alignment of cells to ECM fiber orientation, and (3) a hypothetical role of ECM anisotropy in mechanical cell–ECM reciprocity.

First, certain cell types, such as endothelial cells, fibroblasts, smooth muscle cells, and osteogenic cells, show a monotonic increase in spreading with substrate stiffness. These cells are relatively small on softer substrates, elongate on intermediate substrates, and achieve maximum spreading on highly stiff substrates such as coated glass (Yang et al., 2017). Other cell types, including Jurkat T cells and NIH 3T3 fibroblasts, show a biphasic response of spreading to substrate stiffness, showing maximal cell spreading at an intermediate optimal level of substrate stiffness (Oakes et al., 2018; Wahl et al., 2019; Janmey et al., 2020).

Second, cell alignment is influenced both by the mechanical properties of the fibers and by the cell adhesion properties. In Friedrichs et al. (2007), cells are cultured on a two-dimensional substrate assembled out of thin aligned collagen fibrils. Cells align along the collagen fibrils and bundle parallel fibrils together at their poles and deform the orthogonal fibrils, which creates holes in the substrate. When this experiment was repeated with fragile fibrils, the cell did not elongate, and the fibers surrounding the cell were digested. This suggests that cells require a firm ECM to adhere to and that anisotropic traction force is required to elongate and align to the fibrils. Next, they found that the cell adhesions to the fibrils influence cell alignment. In general, cells adhere to the ECM with integrins, which are membrane-piercing receptors that bind to proteins in the ECM with varying binding strengths, possibly regulated by mechanical tension (Kechagia et al., 2019). Specifically, Friedrichs et al. (2007) found that cells expressing the integrin $\alpha_{2}\beta_{1}$ align on the fibrils, whereas cells that did not express this integrin adhered to the fibrils but did not align.

Finally, cells not only respond to cues in the ECM but also reorient the fibers in the ECM. Contractile breast cancer cells deform fibrous collagen and reorient the fibers to point toward themselves. Pairs of these contractile cells create aligned bridges of fibers between them (Kim J. et al., 2017).

Computational modeling is well-suited for providing insights into mechanical cell–ECM reciprocity (Crossley et al., 2024). Before introducing our own approach, we briefly review a selection of specific computational models of cell–ECM reciprocity involving mechanosensitive adhesions and a fibrous ECM. We highlight two factors that are crucial to be included in a computational model of the reciprocity between ECM fiber alignment and cell morphology, namely, (i) cell-induced changes in ECM structure and (ii) ECM-induced changes in cell shape.

We first review computational models focused on ECM mechanics in response to cell contraction (i). Using a 3D finite element (FE) representation of the ECM, Paukner et al. (2023) showed that cell contractility and force-dependent cell–ECM adhesions suffice for guiding cell migration upward stiffness gradients. This model focused on ECM deformation by the cell but could not capture cell shape change due to changes in the ECM because the cell was modeled as a point particle with an adhesive annulus. They concluded that cell contractility, combined with mechanosensitive cell–ECM adhesions, can explain several phenomena in cell migration. In a different study of cell migration, Feng et al. (2019) introduced a simple bead–spring network approximation of a deformable ECM and a migrating ellipsoidal cell. They showed that a torque balance on the mechanosensitive adhesions of the cell causes the cell to orient along fibers, after which the cell starts migrating. In Feng et al. (2019), the cell’s ability to sense the fiber orientation disappears if the fibers’ bending modulus is too high, showing that in this model, fiber orientation is sensed through mechanical interactions with the fibers. A model that links a fibrous network with breakable cross-links to a circular radial cell suggests that fiber accumulation can enhance cell–ECM adhesion by increasing the number of available binding sites for cellular adhesion (Cao et al., 2017). Altogether, these computational models have studied the potential effect of cell contractility on the ECM, but they did not include the reciprocal effects of the ECM on the cell (ii).

A number of models have considered only (ii), the effect of the ECM on cell behavior. For example, Vargas et al. (2020) showed how different cell migration modes can emerge based on adhesion maturation and stress fiber strength using a 3D finite element model of a moving cell on a non-fibrous, uniformly structured ECM. A different finite element model of cell migration showed how cell deformation and ECM porosity are of primary importance in amoeboid cell migration (Campbell and Bagchi, 2021).

Models combining (i) and (ii), thus closing the loop to full mechanical ECM reciprocity, include those by van Oers et al. (2014), Rens and Merks (2017), and Rens and Merks (2020). In these models, cell shape is modeled using the cellular Potts model (CPM) and coupled to a finite element (FEM) simulation of the ECM to form a hybrid CPM. Early CPM–ECM couplings assumed that cellular protrusions are stabilized on highly stressed substrates (van Oers et al., 2014), showing how mechanical cell–cell communication can play a role in angiogenesis. Subsequently, this coupling was extended by including a comprehensive model of mechanosensitive adhesion between the cell and the ECM, leading to emergent cell spreading, spontaneous cell elongation, and durotaxis (Rens and Merks, 2020). Although these models consider full mechanical reciprocity, their ECM is homogeneous, i.e., there are no fibers. One of the first models of mechanical cell–ECM reciprocity featuring a fibrous ECM was used to explain how bands form between two contractile cells in a fibrous ECM and how the two cells elongate toward each other by the remodeled matrix (see Reinhardt et al., 2013, reviewed in Crossley et al., 2024). Another sophisticated model of cell–ECM reciprocity is that of Kim M. C et al. (2018), where a triangulated dynamic cell is coupled to a fibrous ECM using an FEM simulation. They studied how cells can sense local stiffness in the fiber network by considering filopodia–fiber binding. In this study, we build upon our previously introduced hybrid CPM and molecular dynamics (MD) model (Tsingos et al., 2023). This model approximated the ECM by representing fibers using a beads-and-spring model, where the fibers were linked using cross-linkers. In this work, we modeled how the cell’s contractions form the ECM and how these deformations propagate far into the network. Furthermore, the cells’ contractile forces are counteracted by the ECM, leading to less contraction in a highly cross-linked, stiff ECM and high cellular contraction in a soft ECM. In this model, the cellular adhesions were static, and new adhesions could not be formed with the ECM.

To study the alignment of cells in anisotropic networks, we extend our previous hybrid CPM and molecular dynamics model (Tsingos et al., 2023) with dynamic adhesions. The choice of how to couple cellular morphology and ECM dynamics is delicate as it encodes the biological hypothesis of how cells sense and react with the ECM. In this work, we adopt the coupling between cell and ECM proposed by Rens and Merks (2020) and apply it to a hybrid CPM with discrete fibrous ECM (Tsingos et al., 2023). The coupling is made by assuming that the cell exerts cytoskeletal contraction forces through integrin-based adhesions that behave according to the two-spring model (Schwarz et al., 2006; Doyle et al., 2022). In essence, the two-spring model views adhesion as a mediator between the contractile forces of the cell and the restoring forces of the ECM. The tension on the adhesion builds up slowly on the soft ECM and quickly on the stiff ECM as the cell applies its contractile forces. Additionally, we assume that adhesions strengthen as tension increases. Adhesion strength is quantified by the number of integrin proteins bound to the adhesion. These assumptions, when combined with an isotropic material in a hybrid CPM, are sufficient to produce phenomena such as cell spreading, spontaneous elongation, and durotaxis (Rens and Merks, 2020). We implement this two-spring adhesion model in the hybrid CPM with a discrete fibrous ECM (Tsingos et al., 2023) and use the new model to investigate the reciprocity between fiber orientation and cell morphology. Specifically, with this new fibrous ECM model, we show how cell elongation on oriented gels can be considered a special case of stiffness-dependent cell spreading as fibers are easier to bend than to stretch. Furthermore, we study how cell protrusions can reorient fibers, thereby increasing tension on adhesions and stabilizing the protrusions.

## 2 Methods

### 2.1 Modeling approach

**FIGURE 1:**
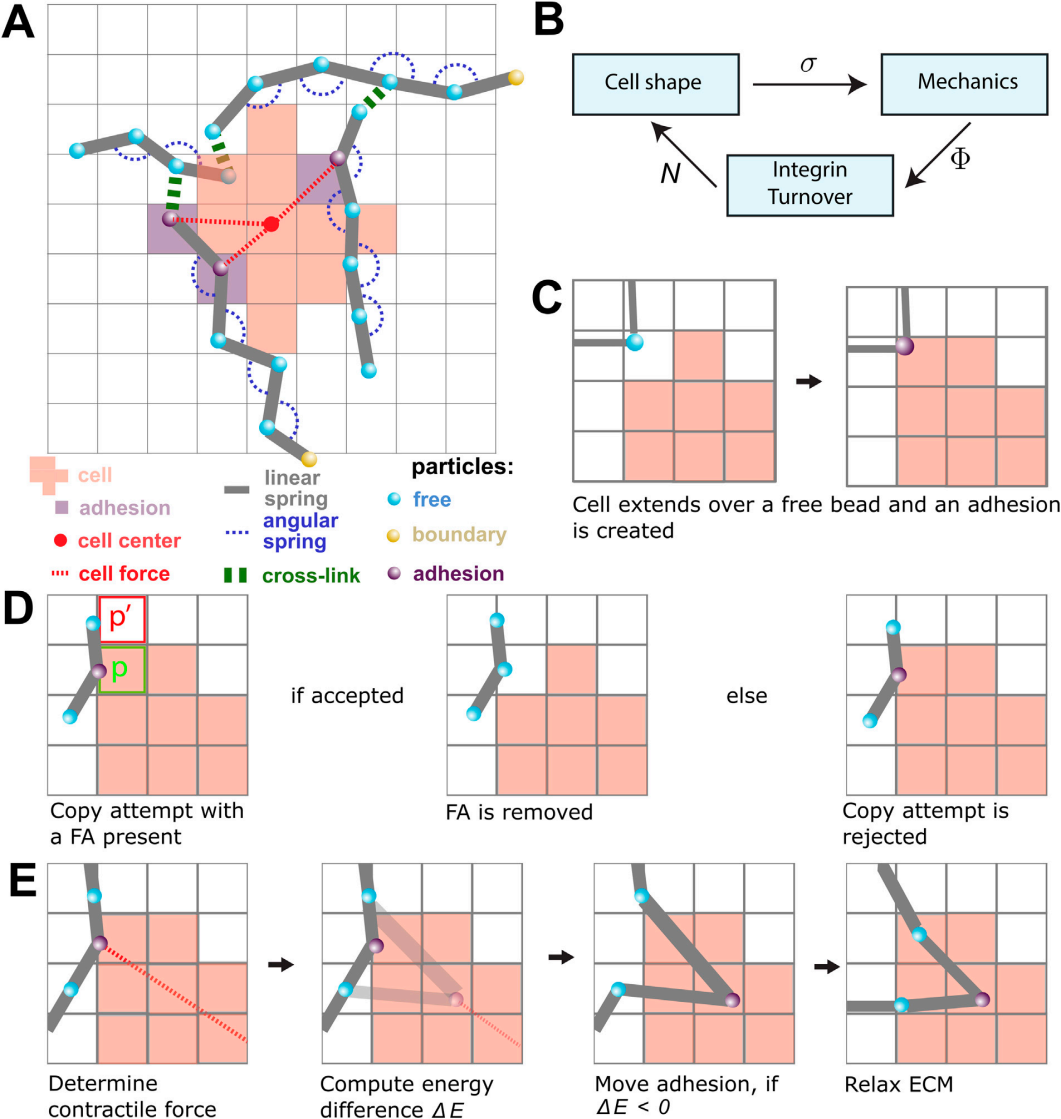
An overview of the model components and the model rules. **(A)** A schematic overview of the components in the model. The lattice-bound cellular Potts cell (red squares) is attached to the beads (cyan) and springs (gray) of the ECM through the focal adhesions (FAs; purple). The contractile force (dashed red) is visualized between FAs and the center of mass of the cell (red point). FAs connect the cell to the strands of the ECM. The strands are cross-linked (green), and particles outside the simulation domain are fixed (yellow). **(B)** The submodels of the model are coupled as shown in this figure. The CPM is responsible for the cell shape and sends the lattice state σ to the mechanical part of the model. The mechanical part computes the tensions Φ in the FAs and gives this to the FA part of the model. The tensions are used to calculate the number of bound integrins N(i) for each FA i . These numbers are then used in the CPM to update the new cell shape. **(C)** A scheme showing the creation of a new FA. When a cell extends over a free bead, it becomes an adhesion bead. **(D)** Scheme showing a retraction copy-attempt when an FA is present. The FA is removed if the copy-attempt is accepted (left). The presence of the FA increases the likelihood of rejecting the copy-attempt (right). **(E)** An FA is pulled toward the center of the cell in a process resembling a CPM copy-attempt: first, the energy change between the original position of the FA and the new FA is determined by only considering springs that are directly attached to the FA. The FA is moved if the energy difference is negative. Finally, the complete ECM is relaxed by running the molecular dynamics simulation.

We have introduced dynamic descriptions of mechanosensitive focal adhesions (FAs) into a hybrid CPM and MD model (Tsingos et al., 2023). The CPM part dynamically describes cell shape changes, and the MD part simulates a cross-linked network of ECM fibers and its dynamical response to cellular forces. In our previous work, the CPM was connected to the MD model through static adhesion particles. In the present model, the buildup and breakdown of FAs are modeled dynamically using an ordinary differential equation model that describes FAs as clusters of integrins, with the breakdown rate assumed to be dependent on the mechanical tension within the FAs. This ordinary differential equation (ODE) model for FAs and their constituent integrins was adapted from the work by Novikova and Storm (2013), as shown in one of our previous models featuring a continuum description of the ECM (Rens and Merks, 2020). Figure 1 provides an overview of the key elements of the model.

The models are coupled using an operator-splitting approach. The three submodels are sequentially iterated to a steady state, where the output state of one submodel is used as the input state for the next submodel (Figure 1B). The simulations were run until a quasi-steady state was reached, i.e., until no large further changes were observed. In the remainder of this section, we will describe each of the three submodels and the coupling strategies.

### 2.2 Cellular Potts model

To describe cell shape changes, we employ the cellular Potts model (Graner and Glazier, 1992; Hirashima et al., 2017). The CPM is a lattice-based model in which cell shape is defined as a collection of connected lattice sites. We implemented a CPM on a square grid $\Lambda \subseteq Z^{2}$ of 200×200 lattice sites. Each lattice site $\vec{x}\in \Lambda$ is assigned a spin $\sigma \left(\vec{x}\right)\in Z_{\geq 0}$ , which defines a spin field $\sigma :\Lambda \to Z_{\geq 0}$ . The collection of connected lattice sites that have the same positive spin n defines the shape of the cell n . As shown in Figure 1A, the red lattice sites indicate the shape of a single cell. The set of lattice sites with spin 0 is not occupied by a cell.

The CPM evolves through a sequence of random extensions and retractions, whose probability is given by a balance of contractile and extensile forces and forces due to adhesion with the ECM. These are given by a Hamiltonian energy function.

$$H\left(\sigma \right)=\lambda A^{2}+\underset{\vec{x}\in \Lambda }{\sum }\underset{{\vec{x}}^{\prime }\in \text{NB}\left(\vec{x}\right)}{\sum }J_{\sigma \left(\vec{x}\right)\sigma \left({\vec{x}}^{\prime }\right)}1_{\sigma \left(\vec{x}\right)\neq \sigma \left({\vec{x}}^{\prime }\right)}-\lambda_{c}\frac{A}{A+A_{h}},$$ (1)

where $A=|\left\{\vec{x}\in \Lambda :\sigma \left(\vec{x}\right)>0\right\}|$ is the area of the cell, $\text{NB}\left(\vec{x}\right)$ is the set of lattice sites in the neighborhood of $\vec{x}$ , and λ , J , $\lambda_{c}$ , and $A_{h}$ are parameters. The first part of Equation 1 describes the contractility of the cell with magnitude λ . The second term penalizes, with strength J , interfaces between the cell and the medium, effectively creating a line tension along the cell’s perimeter. The final term describes the formation of non-integrin-based adhesions with the substrate, which bind with a strength parameter $\lambda_{c}$ and a saturation parameter $A_{h}$ .

The Hamiltonian is minimized through Metropolis dynamics, as previously described by Graner and Glazier (1992), thus dynamically updating the cell’s shape. In brief, we iteratively select a random lattice site $\vec{x}$ and a random adjacent lattice site ${\vec{x}}^{\prime }$ . We then calculate the energy difference ΔH that would result due to the update and accept the copy attempt with probability P(ΔH)=1 if ΔH≥0 , and P(ΔH)=exp(−ΔH/T) for ΔH>0 , where T is a cell motility parameter.

The acceptance probability of a copy attempt is determined by the energy change:

$$\Delta H=H\left(\sigma \left({\vec{x}}^{\prime }\right)\right)-H\left(\sigma \left(\vec{x}\right)\right)+\Delta H_{\text{FA}},$$ (2)

where $\Delta H_{\text{FA}}$ is an additional penalty for breaking integrin bonds that the cell might have with the ECM at that location.

The term $\Delta H_{\text{FA}}$ in Equation 2 is non-zero only if the copy attempt corresponds to a retraction from a site $\vec{x}\in \Lambda$ that contains an FA, and in this case,

$$\Delta H_{\text{FA}}=\lambda_{\text{FA}}\frac{\underset{N\in N\left(\vec{x}\right)}{\sum }\left(N-N_{0}\right)}{\underset{N\in N\left(\vec{x}\right)}{\sum }\left(N-N_{0}\right)+N_{h}},$$

where $N\left(\vec{x}\right)$ represents the number of integrins in the FAs situated at $\vec{x}$ , $\lambda_{FA}$ represents a scaling parameter, $N_{0}$ represents the initial size of an FA, and $N_{h}$ represents a saturation parameter. If a copy attempt is accepted that leaves an FA outside of the cell, the FA is removed, as shown in Figure 1D. If a copy attempt is accepted that extends over a free bead of the ECM, then a new FA is created, as shown in Figure 1C.

### 2.3 Extracellular matrix model

The ECM is described as a set of fibers connected through cross-linkers, forming a fiber network that is superimposed on the CPM lattice (Figure 1A). A fiber is built out of $n_{\text{beads}}$ beads, which are linked together with springs of stiffness $K_{\text{polymer}}$ and rest length $r_{\text{polymer}}$ . Fibers are illustrated in Figure 1A, where the blue beads are connected by gray springs to form different fibers. Next to springs linking beads into fibers of contour length $r_{\text{polymer}}\cdot \left(n_{\text{beads}}-1\right)$ , consecutive triplets of beads in a fiber are connected with a harmonic potential with bending rigidity $K_{\text{bend}}$ . This angular constraint ensures that unforced fibers remain straight and are illustrated with dashed blue curves in Figure 1A. To create a network, cross-linkers are added to the fibers (the green dashed lines in Figure 1A). Cross-links are defined as springs with a small rest length and stiffness equal to $K_{\text{cross}}=K_{\text{polymer}}$ and link different fibers together to form a connected network.

To create a fiber network, we followed the method introduced by Tsingos et al. (2023) with small modifications for creating networks of aligned fibers. In brief, we distributed $N_{\text{strands}}$ randomly and uniformly in space, selecting fiber orientations from the von Mises distribution to control the degree of fiber alignment. Fibers were created as follows: the position of the middle bead ${\vec{b}}_{k}\in \Lambda$ with $k≔\text{floor}\left(n_{\text{beads}}/2\right)$ of a strand was selected at random from a uniform distribution, and a random angle θ∈[0,2π) was selected from the von Mises distribution with $\mu \in \left[0\right.,\left.2\pi \right)$ and $\kappa \in \left[0\right.,\left.\infty \right)$ . Then, the remaining positions ${\vec{x}}_{i}$ making up the beads were defined via

$${\vec{b}}_{i}={\vec{b}}_{k}+\left(i-k\right)r_{\text{polymer}}\vec{v},\;\text{for }i\in \left\{0,\ldots ,n_{\text{beads}}-1\right\},$$

where v=(cos⁡θ,sin⁡θ) is a unit vector with angle θ . Constructing the fiber positions in this way ensures that the middle of each fiber is within the simulation domain, while only the endpoints might extend beyond the simulation domain. After the fibers have been introduced, the network is cross-linked as described in the previous work by Tsingos et al. (2023).

The springs connecting pairs of beads and the bending rigidity connecting triples of beads impose forces on the ECM and make the fiber network dynamic. The positions of the beads ${\vec{b}}_{1}\left(t\right),\ldots ,{\vec{b}}_{n}\left(t\right)\in R^{2}$ are governed by the overdamped Langevin equation of motion

$$\gamma_{\text{drag}}\frac{d}{dt}{\vec{b}}_{i}={\vec{F}}_{i}+{\vec{W}}_{i},$$ (3)

where $\gamma_{\text{drag}}$ is a drag coefficient, $F_{i}$ is the force on the i th particle, and ${\vec{W}}_{i}$ is a random force satisfying $\left\langle {\vec{W}}_{i}\right\rangle =0$ and $\left\langle {\vec{W}}_{i}^{2}\right\rangle =2\gamma_{\text{drag}}T_{\text{ECM}}/\Delta t$ with $T_{\text{ECM}}$ representing a parameter for degree of noise in the system and Δt representing the size of a timestep. Equation 3 was integrated to a steady state, with fixed Δt during the simulation using the HOOMD-blue molecular dynamics library (Anderson et al., 2020).

The energy of a single spring with a rest length $r_{0}$ and spring constant k , connecting a pair of beads (i,j) , is determined by the potential:

$$U_{ij}=\frac{k}{2}\Delta r_{ij}^{2},\;\text{where}\;\Delta r_{ij}=r_{0}-\|b_{i}-b_{j}{\|}_{2},$$

where $\|\left(x,y\right){\|}_{2}=\sqrt{x^{2}+y^{2}}$ is the Euclidean norm. Similarly, the harmonic potential between a triple of beads (i,j,k) is defined as

$$U_{\mathrm{ijk}}=\frac{K_{\text{bend}}}{2}\Delta \theta ,\;\text{where}\;\Delta \theta_{\mathrm{ijk}}=\text{shortest angle between }{\vec{b}}_{i},{\vec{b}}_{j},{\vec{b}}_{k}.$$

where $K_{\text{bend}}$ is the bending rigidity. Some beads are fixed in space and are excluded from Equation 3. These beads are at the boundary of the system, effectively clamping the ECM at the sides of the integration domain.

### 2.4 Focal adhesions

FAs, schematically shown as purple beads in Figure 1A, are modeled as clusters of catch-slip bonds (Novikova and Storm, 2013; Rens and Merks, 2020; Schwarz et al., 2006). Each cluster is assumed to be in constant flux as integrins are added and removed from the cluster. The integrin addition rate is independent, whereas the removal rate is suppressed by mechanical tension due to the contractile force of the cell and the restoring force from the ECM. The number of integrins N in a single focal adhesion is the *size* of that FA and changes when under tension Φ following the equation:

$$\frac{dN}{dt}=\gamma \left(N_{\text{tot}}-N\right)-d_{0}d\left(f_{*}\frac{\Phi }{N}\right)N,$$ (4)

where γ is the binding rate of integrins, $N_{\text{tot}}$ is the maximum number of integrins in a single FA (Changede and Sheetz, 2017), $d_{0}$ is the base detachment rate, $f_{*}$ is the force scale, and d(φ) is a function of the tension per integrin that encodes the response of mechanical tension to the unbinding rate of a single integrin. We use a model for a catch-slip integrin that takes the following form:

$$d\left(\varphi \right)=e^{\varphi -\phi_{s}}+e^{\phi_{c}-\varphi },$$

where $\phi_{s}$ and $\phi_{c}$ describe the slip and catch regime of an integrin, respectively (Novikova and Storm, 2013; Rens and Merks, 2020).

The tension Φ on the FAs is due to the force balance of the contractile cellular force and the resultant force from the ECM. To calculate the contractile force, we assume that the cell’s cytoskeleton applies a force proportional to the distance from the cell center (Lemmon and Romer, 2010), effectively modeling the cytoskeleton as a spring connecting each FA to the cell’s center, as sketched in Figure 1A with the dashed red lines connecting purple adhesion particles to the cell center. The energy that the cytoskeleton exerts is then assumed to be $E_{\text{cyto}}=\sum_{\vec{x}}\frac{K_{\text{cyto}}}{2}{\left(\vec{x}-{\vec{x}}_{\text{center}}\right)}^{2}$ , where $\vec{x}$ is the position of an FA, ${\vec{x}}_{\text{center}}$ is the center of the cell to which the FA belongs, and $K_{\text{cyto}}$ is a spring constant encoding the isotropic cell force. Similarly, the energy of parts of the ECM that are directly linked to the FA is defined as $E_{\text{ecm}}$ . An FA is then displaced using an algorithm that is similar to the CPM algorithm: We attempt to move an FA one lattice site toward the cell center, and this movement is accepted if it is energetically favorable. Otherwise, the movement is rejected. Specifically, we first compute the total energy $E=E_{\text{cyto}}+E_{\text{ecm}}$ . Then, the energy $E^{\prime }$ is computed if the FA was moved one lattice site toward the center of the cell. If the difference $E^{\prime }-E$ is negative, the FA is moved to the new position; otherwise, it is kept in place. This means that FAs are moved independently from each other and can lead to multiple FAs, occupying the same lattice site. Furthermore, the energies E are independent of Equation 1, which describes the energy of the cell.

### 2.5 Parameter values

Table 1 lists the parameter values used for the CPM and MD models. They are dimensionless and require scaling to fit to measurable quantities. We follow the previous work by Tsingos et al. (2023) for this scaling, and we briefly summarize the main points in this section. A single lattice site of the CPM is set equal to 0.25μm×0.25μm , and $10^{4}$ model timesteps is roughly 8 h. The CPM parameters λ , J , $\lambda_{c}$ , T , and $A_{h}$ are calibrated to show generic cell area and activity in the absence of the ECM, and the FA parameters γ , $d_{0}$ , and $f_{*}$ were estimated such that the final FA size distribution was wide enough to differentiate between softer and stiffer parts of the ECM. As in our previous work, we fit the force units of the model to match up on the widely varying tensile modulus Y of collagen. We set $Y=10^{6}\;Pa$ , which yields a spring constant of $3.1\cdot 10^{-2}\;{Nm}^{-1}$ . As described by Tsingos et al. (2023), tensile modulus is converted into the spring constant by approximating a single collagen fiber as a cylindrical rod of diameter 0.125μm and applying the formula K=YA/L , with A representing the cross-sectional area and L representing the length of a collagen segment. This choice results in a contraction force of $3.1\cdot 10^{-4}\;{Nm}^{-1}$ , leading to typical traction forces ranging from 8nN to 15nN on a single FA, with a total range of $10^{-8}$ to $10^{-10}\;N$ . These values are slightly lower than those reported by Tsingos et al. (2023) but fall in the correct order of magnitude for cell traction forces (Wakatsuki et al., 2000; Labernadie and Trepat, 2018). Although most parameters in this model were estimated to produce reasonable behavior, the predicted dynamics and interactions provide insights into the role of mechanical reciprocity in cell biology.

**TABLE 1:** Parameter values used in the simulations unless otherwise specified. The parameter values reported here are the scaled values, which is why they may appear as non-rounded numbers despite being chosen values.

Symbol	Value	Unit	Reference
λ	$4.96\cdot 10^{7}$	${\text{Nm}}^{-3}$	Estimated
J	$9.30\cdot 10^{-3}$	${\text{Nm}}^{-1}$	Estimated
$\lambda_{c}$	$3.87\cdot 10^{-13}$	Nm	Estimated
$A_{\text{h}}$	50.0	$\mu {\text{m}}^{-2}$	Estimated
$\lambda_{\text{FA}}$	800	−	Estimated
$K_{\text{cyto}}$	$3.10\cdot 10^{-4}$	${\text{Nm}}^{-1}$	Estimated
T	50	−	Estimated
$T_{\text{ECM}}$	0.001	−	Tsingos et al. (2023)
γ	2.88	${\text{s}}^{-1}$	Estimated
$d_{0}$	$2.88\cdot 10^{-2}$	${\text{s}}^{-1}$	Estimated
$f_{*}$	$1.29\cdot 10^{10}$	${\text{N}}^{-1}$	Estimated
$\phi_{s}$	4.02	−	Novikova and Storm (2013)
$\phi_{c}$	7.76	−	Novikova and Storm (2013)
$N_{\text{tot}}$	390	−	Changede and Sheetz (2017)
K	$3.10\cdot 10^{-2}$	${\text{Nm}}^{-1}$	Estimated
$K_{\text{bend}}$	$3.88\cdot 10^{-15}$	${\text{Nmrad}}^{-2}$	Estimated
$\theta_{0}$	3.14	rad	Fibers are preferentially straight
Fiber density	0.48	$\mu {\text{m}}^{-2}$	Estimated
Fiber anisotropy κ	0, 10	—	Estimated based on the resulting order parameter
$\text{NB}\left(\vec{x}\right)$	Second-order/Moore	—	Estimated

### 2.6 Statistical significance

**FIGURE 2:**
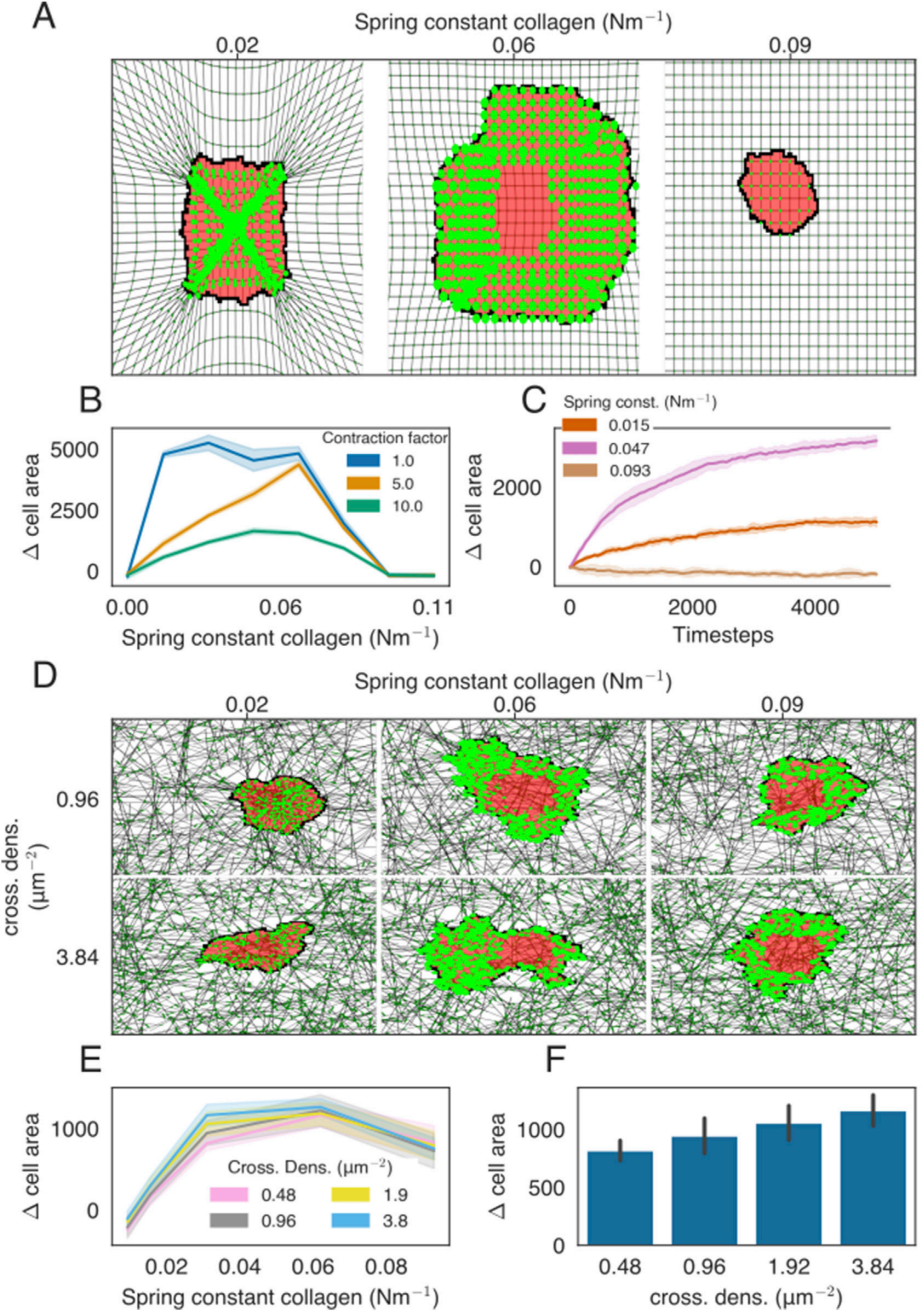
Cell spreading on isotropic ECMs with regular or network structures. **(A)** Example of cell spreading on an isotropic ECM for three different spring stiffness values. The cell is colored in red, FAs are presented as bright-green discs, ECM strands are in gray, and ECM crosslinks are shown in dark green. The radius of the bright-green discs is proportional to the size of the FAs. All FAs are assigned to only a single lattice site, even if the visualization may suggest otherwise. **(B)** Difference in the cell area from the starting size for the ECM of different stiffness. Colors indicate the factor by which the contraction force of the cell is multiplied. **(C)** Time-evolution of the cell area as a function of ECM spring stiffness. **(D)** Simulation snapshots of cell spreading on the isotropic ECM for a range of spring constants and cross-link densities. **(E)** Difference in the cell area from the starting size, at t=0 , for ECM of different stiffnesses. Colors indicate the cross-link density. **(F)** Bar graph showing the final cell size as a function of collagen density, with stiffness parameter $K=0.031{Nm}^{-1}$ .

**FIGURE 3:**
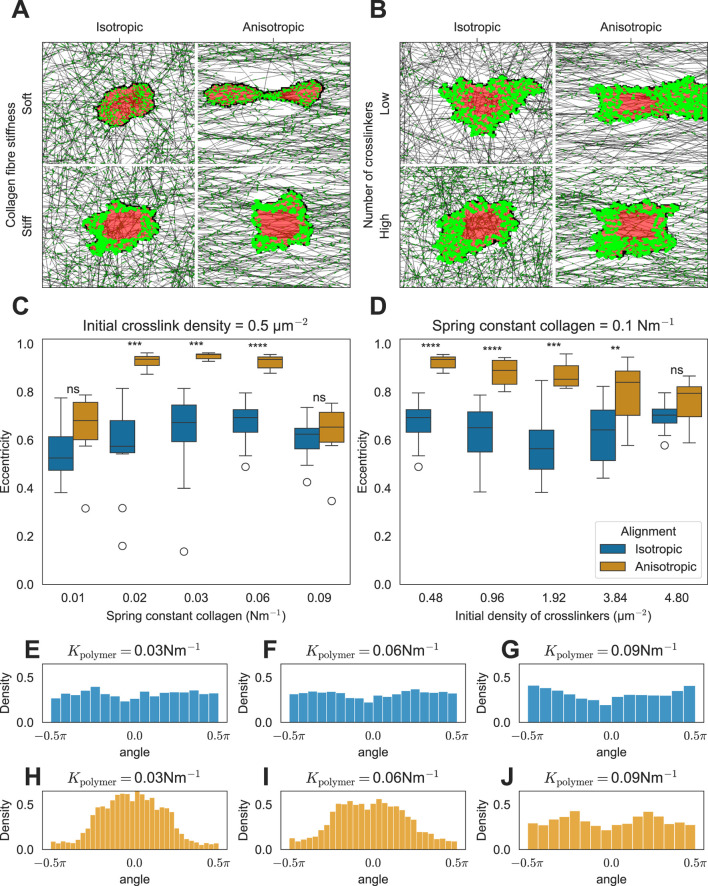
Effect of ECM anisotropy on the cell shape. **(A)** Snapshots of simulations showing the effect of matrix anisotropy on the cell shape as a function of collagen stiffness (K = $0.016{\text{Nm}}^{-1}$ (soft) and $K=0.093{\text{Nm}}^{-1}$ (stiff)). **(B)** Snapshots of simulations showing the effect of matrix anisotropy on the cell shape as a function of cross-linker density ( $\left[N_{\text{cross}}\right]=0.48\;\mu {\text{m}}^{-2}$ (low) and $\left[N_{\text{cross}}\right]=4.8\;\mu {\text{m}}^{-2}$ (high)). **(C)** Distribution of cell eccentricities as a function of collagen stiffness ( K ). Distribution of cell eccentricities as a function of cross-linker density **(D)**. **(E–J)** Distributions of FA angles relative to the horizontal axis passing through the cell’s center of mass are shown for isotropic ECM conditions **(E–G)** and anisotropic ECM conditions **(H–J)**.

**FIGURE 4:**
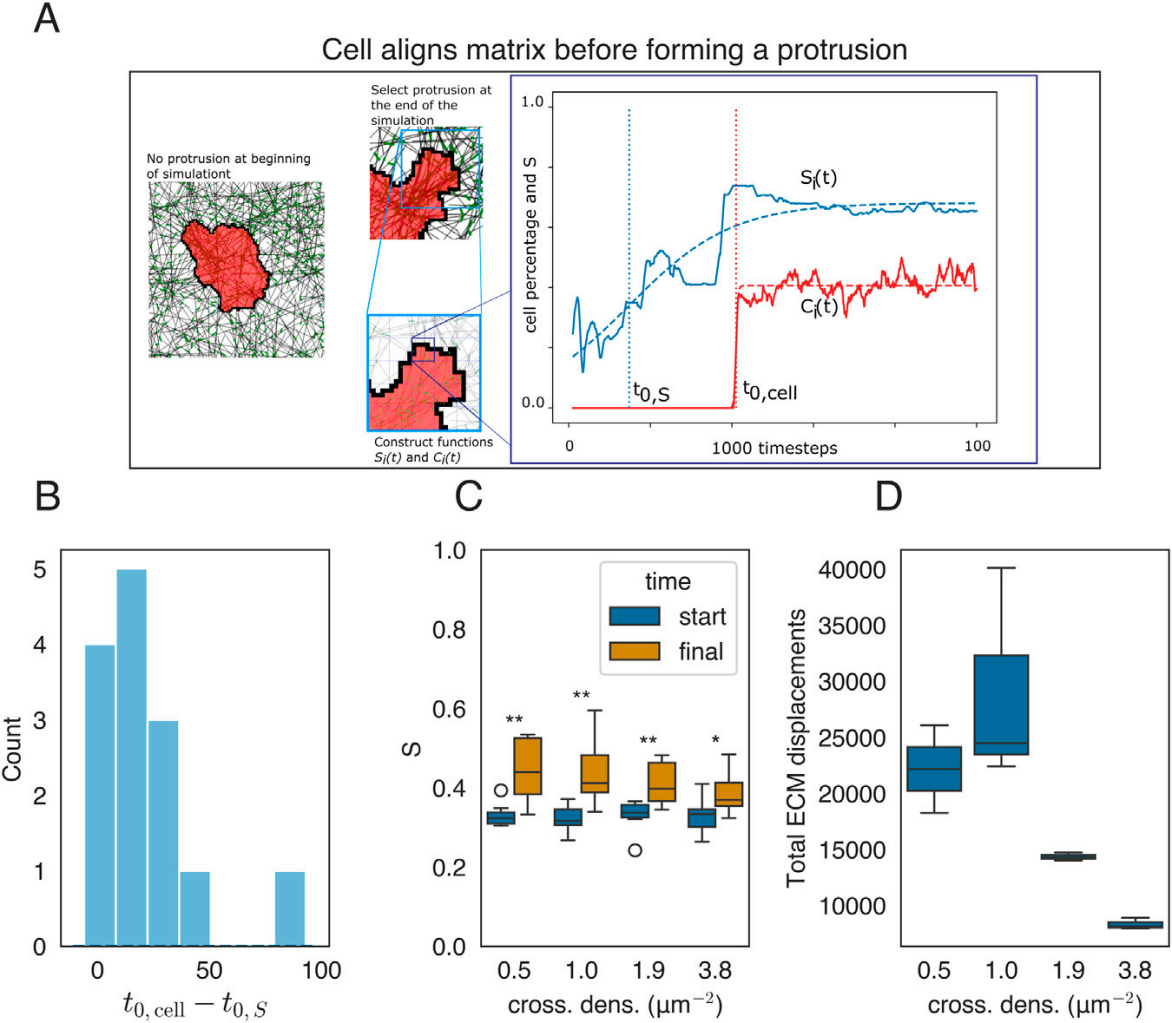
Mechanical reciprocity between local cell spreading and local ECM alignment. **(A)** Cell state at two time points of the simulation corresponding to t=0 and t=1000 in the far right panel. An example of the time series $S_{i}\left(t\right)$ (blue) and $C_{i}\left(t\right)$ (red) for a specific bin i together with a fitted sigmoid function is included (dashed line), and the center of the fitted sigmoids is indicated on the x -axis. **(B)** Distribution of the difference in time of ECM alignment and cell spreading. Almost all the mass is on the positive x -axis, indicating that the ECM reorientation occurs before the cell spreads. **(C)** The average order parameter $S_{i}\left(t\right)$ of the network in an annulus around the cell at t=0 and t=1000 . **(D)** Total displacement of ECM beads as a function of cross-link density.

The error bars shown in Figures 2–4 denote the mean ±1 standard deviation. Statistical significance: we computed a p -value using the Welch’s test, which we reported with the following symbols: (ns) p≥0.05 , (*) p<0.05 , (**) p<0.01 , (***) p<0.001 , and (****) p<0.0001 .

## 3 Results

### 3.1 Stiffness-dependent cell spreading

Cell spreading emerges in models of mechanosensitive dynamic FAs with a uniform isotropic ECM (Rens and Merks, 2020), suggesting that we should be able to capture cell spreading in the present model as well. To stay close to the simulated case of the isotropic linear elastic material used previously, we first studied cell spreading on a homogeneous matrix constructed by creating long vertical and horizontal strands and cross-linking them at the intersections. On this homogeneous matrix, the cell’s spreading area depends on matrix stiffness in a biphasic manner. Up to an optimum stiffness, the cell-spreading area increases with matrix stiffness, after which the cell spreading area decreases with matrix stiffness (Figures 2A, B; Supplementary Videos S1, S2). After this optimum stiffness, the tension within the FAs reaches the slip regime of the integrins within the FAs. This biphasic effect was not observed in the previous model with a uniform ECM (Rens and Merks, 2020) or in endothelial cells (Reinhart-King et al., 2005). However, such a biphasic response of cell spreading to matrix stiffness was observed in fibroblasts and T-cells (Oakes et al., 2018; Wahl et al., 2019). Interestingly, the biphasic effect disappears when they increase the lifetimes of the integrin–ECM bonds (Oakes et al., 2018). To test whether our model is consistent with this experimental observation, we doubled the slip parameter $\phi_{s}$ , which leads to an increased integrin lifetime, and observed that the biphasic effect shifts to higher stiffnesses (Supplementary Figure S1). We did not study the effect of an increased integrin ligand density on cell spreading as we have not modeled individual integrins. We next tested how contraction force affects cell spreading. Consistent with experiments showing that inhibition of myosin increases cell spreading (Wakatsuki et al., 2003), in our model, we find that cell spreading decreases with an increase in the cell contraction force (Supplementary Figure S2; Supplementary Video S3).

In Rens and Merks (2020), a mechanism based on mechanosensitive FAs was found to explain cell spreading, cell elongation, and durotaxis on a homogeneous, regular ECM. Having implemented a conceptually similar model, we discovered the relationship between ECM stiffness and cell spreading on a homogeneous, regular ECM. Next, we studied the relationship between ECM stiffness and cell spreading on an inhomogeneous, randomized, but isotropic ECM. We constructed isotropic randomized matrices, as described in Section 2.3, by distributing elastic strands of roughly one cell length and cross-linking them together. We observed a biphasic effect on cell spreading when increasing the spring stiffness of the network (Figure 2E; Supplementary Videos S4, S5). We also modified ECM stiffness by changing the cross-link density (Figure 2F). The cell spreading area also increased with cross-linker density, which is consistent with 2D *in vitro* experiments (Mullen et al., 2015).

### 3.2 Cells elongate on the anisotropic matrix due to the local anisotropy of stiffness cues

*In vitro*, cells extend along fibers in the substrate (Lai et al., 2012; Friedrichs et al., 2007; Sapudom et al., 2023; Chaubaroux et al., 2015). We hypothesize that the mechanism driving such elongation is similar to the mechanism of stiffness-dependent cell spreading on a regular, isotropic ECM described in the previous section. We, therefore, asked how ECM isotropy affects cell spreading. Figures 3A, B show example simulations on inhomogeneous matrices with and without anisotropy. Cells placed on anisotropic matrices elongate along the axis of anisotropy, provided that the fibers are not excessively stiff (Figures 3A, C) and that the network is not overly cross-linked (Figures 3B, D). This phenomenon occurs because FAs stabilize rapidly under increased tension. In particular, in the ECM composed of parallel fibers, the difference in tension built up along or orthogonal to the fibers is large as fibers resist extension more effectively than bending. Consequently, FAs stabilize more rapidly in response to force along the ECM compared to force along the orthogonal orientation, resulting in the observed cell alignment. Increasing the bending modulus of ECM fibers leads to the creation of cellular protrusions orthogonal to the ECM orientation (Supplementary Figure S3A). However, these protrusions are small, and the cell still aligns with the ECM fibers (Supplementary Figure S3B).

Consistent with *in vitro* observations, our model simulations predict that FAs form preferentially at the poles of the cells (Figures 3A, B) (Friedrichs et al., 2007; Chaubaroux et al., 2015; Lai et al., 2012). The angles between the cell elongation axis and the FAs are roughly uniformly distributed on isotropic matrices (Figures 3E–G). By contrast, on anisotropic matrices, the FAs are centered at the poles of the elongated cell (see single peaks at approximately 0 radians in Figures 3H, I). This effect was independent of collagen fiber stiffness. For the highest stiffness tested, cells no longer elongated, and FAs become uniformly distributed around the cell (Figure 3J). Similarly, if we increased the degree of cross-linking in anisotropic ECM with intermediate fiber stiffness, the cells failed to elongate (Figures 3B, D; Supplementary Videos S8, S9). In both cases, the fibers provided more resistance to contraction forces perpendicular to the axis of anisotropy than in the low-stiffness case, either due to increased fiber stiffness or increased cross-linking. As a result, the tension in the FAs, pulling perpendicular to the axis of anisotropy, became sufficiently strong for FA maturation, leading to cell rounding. Next, we tested the behavior of the model on strongly cross-linked ECM consisting of stiff fibers. In this situation, our model predicts that cells orient perpendicularly to the axis of ECM anisotropy (Supplementary Figure S4; Supplementary Video S10) because the FAs along the direction of fiber orientation enter the slipping regime and break. Hence, the cell elongates orthogonal to the fiber orientation. Finally, we looked at how the degree of isotropy (as quantified by the order parameter S , with S=0 for full isotropy and S=1 for full anisotropy, see supplemental material) affects the cell shape. The cell eccentricity increased monotonically as a function of the order parameter S (Supplementary Figure S5). In short, we showed that ECM isotropy influences cell morphology as the ECM determines local stiffness perceived by the cell.

### 3.3 Cell remodels fibers before it starts spreading

In the previous sections, we studied how static, global anisotropies of the ECM can affect cell shape. We next looked at potential cell shape changes due to mechanical reciprocity between the cell and the local ECM, potentially driving local ECM anisotropy. We studied the time evolution of the local order parameter $S_{i}\left(t\right)$ and compared it with local cell spreading $C_{i}\left(t\right)$ around an area of a cellular protrusion; both functions are defined below.

The functions $S_{i}\left(t\right)$ and $C_{i}\left(t\right)$ are defined as follows: first, we subdivided the domain into square bins of 5×5 lattice sites. Then, for each bin i , we compute the order parameter $S_{i}\left(t\right)$ for the fibers in that bin and quantified the degree of cell spreading $C_{i}\left(t\right)$ as the ratio of the number of lattice sites belonging to the cell and the total number of lattice sites in the bin (i.e., 25). We perform smoothing of the functions $S_{i}\left(t\right)$ and $C_{i}\left(t\right)$ by computing a moving time average over the past 10 time steps. Next, we use the functions $S_{i}\left(t\right)$ and $C_{i}\left(t\right)$ to study ECM remodeling by the cell.

Figure 4A shows two states of a model simulation, one shortly after initialization and one after $10^{4}$ timesteps, showing a large protrusion at the upper right side of the cell. To quantify the degree of cell spreading and ECM alignment in this region, we selected the square-shaped region around the protrusion of size 20×20 lattice sites (i.e., 16 bins) and studied the local ECM alignment $S_{i}\left(t\right)$ and local cell spreading $C_{i}\left(t\right)$ . An example graph for a single bin is shown alongside the screenshots in Figure 4A, with the order parameter S shown in blue and the degree of cell spreading shown in red. These graphs depict distinct low and high states, with a pronounced transition between them. To examine the variations in the onset of the timing of these transitions, we fitted a sigmoid function to each graph:

$$f\left(t\right)=\frac{L}{1+\exp\left(-k\left(t-t_{0}\right)\right)},$$

and plotted the distribution of $t_{0,\text{cell}}-t_{0,S}$ in Figure 4B, where $t_{0,\text{cell}}$ and $t_{0,S}$ represent the onset times of cell spreading and matrix alignment obtained from the fit, respectively. Most of the mass in this distribution is positive, indicating that the cell remodels the matrix first and then spreads over the remodeled fibers. This behavior disappears if the cell contractile force is reduced (Supplementary Figure S6).

The cells not only remodel the ECM at the pseudopodia but also seem to remodel the ECM all around the cell. We, therefore, quantified the alignment around the cell by taking the average of order parameters in an annulus around the cell given by

$$\left\{S_{i}:r<\|x_{i}-c\|<1.5r\right\},$$

where $S_{i}$ is the order parameter at the end of the simulation in bin i , $x_{i}$ is the middle of bin i , c is the center of the cell, and r is the length of the cell. Figure 4C shows that the average order parameter around the cell increases over time and that cross-linking influences the extent of realignment as higher cross-linker densities decrease the impact of remodeling of the ECM. Additionally, a biphasic relation is observed between the cross-link density and final matrix displacements. Figure 4D presents the average displacements of the matrices after $10^{4}$ timesteps for varying cross-link densities. Fiber networks with a low cross-link density show less displacement compared to those with a medium number. Finally, highly cross-linked fiber networks again show reduced displacements. This biphasic relation between matrix cross-linking and matrix displacement becomes evident when considering two extreme cases: an ECM with few cross-links and an ECM with many cross-links. In the case of an ECM with few cross-links, the displacements induced by the cell do not propagate effectively through the matrix, resulting in low overall displacement. Conversely, in a highly cross-linked ECM, the force required to displace the matrix exceeds the cell’s contractile force, thereby leading to minimal displacement. This effect of cross-linking on displacement can also be explained using the concept of percolation of the network. Low cross-linking leads to a less connected and non-percolated network, i.e., not all fibers are connected to one another (Casey et al., 2021), and the networks considered in this paper are percolated when the cross-link density is higher than $1.0\;\mu {\text{m}}^{-2}$ (Supplementary Figure S8).

## 4 Discussion

In this paper, we studied the effect of mechanical cell–matrix reciprocity across different ECMs. Assuming that tension-mediated integrin turnover drives FA maturation, we find that FA formation depends on both the angle of the ECM fibers with the cell and the cell’s contractile force. Since FAs determine where the cell adheres to the ECM, we see how ECM anisotropy influences cell morphology. In the isotropic ECM, where fibers are uniformly oriented, FAs form evenly around the cell, leading to uniform cell spreading over the fibers. On the anisotropic ECM, however, FAs preferentially form parallel to the fibers, causing the cell to elongate and align with the overall orientation of the fibers. Interestingly, this model also predicts a mechanical reciprocity between cell contractility and ECM anisotropy: the cell’s contractile forces reorient ECM fibers toward itself, enhancing the cell’s ability to adhere more strongly as it continues to spread over these newly aligned fibers. In this section, we discuss these observations and link them to existing experiments and models. We start with the similarities and differences of this model with earlier work that studies the mechanosensitivity of FAs on a homogeneous isotropic matrix.

The model proposed in this paper is not a strict improvement over the conceptually similar model proposed by Rens and Merks (2020), which uses a continuum approach to model a homogeneous isotropic ECM. Instead, the type of ECM considered in both models is different; for ECMs with small physical components, a continuous approach, as described by Rens and Merks (2020), could be better suited, whereas many other realistic ECMs require the added plastic, fibrous details that this paper develops. In the current paper, a cell situated on a fibrous and non-elastic ECM, such as collagen, is modeled, whereas Rens and Merks (2020) considered an elastic ECM, such as a polyacrylamide gel. Both models explain how cells spread less on soft substrates and more on stiffer substrates. However, in the model proposed by Rens and Merks (2020), the cell starts elongating on substrates of intermediate stiffness, whereas this spontaneous elongation is not observed in the currently discussed model. A possible reason for this discrepancy is the additional assumption made by Rens and Merks (2020), namely, that planar substrate stress strengthens FAs. To what extent FAs strengthen due to substrate stress in the context of a fibrous ECM remains unclear.

The model presented in this paper predicts a biphasic relationship between the extent of cell spreading and substrate stiffness: on an isotropic ECM, the cell spreads up to an optimum stiffness, whereas for even stiffer substrates, the cell area decreases again (Figure 2). Cell spreading on an isotropic regular ECM is best compared with *in vitro* spreading of cells on a hydrogel, as is found in the biphasic spreading of T cells (Oakes et al., 2018; Wahl et al., 2019). In the presented model, the biphasic relation arises from the catch-slip behavior of the integrins in the FAs, Equation 4, regulated by the slip parameter $\varphi_{s}$ : on soft to intermediate stiffness ECMs, the tension on each integrin φ is less than $\varphi_{s}$ , leading to higher spreading, whereas on stiff ECMs, the FAs enter the slip-regime as $\varphi >\varphi_{s}$ , leading to less spreading. This mechanism, suggested by Oakes et al. (2018), is contrasted by Wahl et al. (2019), who suggested that it is not the mechanosensitive integrins that lead to the biphasic relation but rather a different mechanosensitive protein linking the cell’s actin cytoskeleton to the ECM receptor. To study this different mechanism, a different description of cellular contractility and FA dynamics could be implemented. Other cell types, such as endothelial cells, spread monotonically on increasing ECM stiffness (Reinhart-King et al., 2005). Monotonic cell spreading occurs in our model if we increase the slip regime parameter $\varphi_{s}$ of the integrin, effectively replacing the catch-slip integrin with a pure catch integrin (Supplementary Figure S1).

After considering cell spreading on isotropic and homogeneous ECMs, we subsequently studied cell spreading on isotropic but inhomogeneous ECMs, which model certain types of *in vitro* fiber networks such as collagen or fibrin networks. The mechanics of fibrous networks are different from those of homogeneous ECMs because both respond differently to stress due to, for example, the possibility of sliding and reorientation of fibers (Storm et al., 2005). Our model predicts an increase in cell area with an increase in cross-link density, which aligns with findings that osteogenic cells are larger on highly cross-linked collagen fiber networks than on low cross-linked networks (Mullen et al., 2015). In a contrasting study, Baker et al. (2015) compared the cell spreading of NIH 3T3 fibroblasts and human MSC cells on synthetically produced fiber networks with low or high fiber stiffness. The cells spread more on softer fibers and less on stiffer fibers. Together with a computational follow-up study, a model emerged where cells on soft fibers could pull additional fibers toward the cell over which it could spread (Baker et al., 2015; Cao et al., 2017). To study this effect in our model, it can be extended to include two additional mechanisms that are included in the study by Cao et al. (2017). First, FAs should strengthen when the fiber density is higher. Second, cross-links break under stress, leading to higher fiber recruitment in soft networks.

We next studied the effect of anisotropy in the ECM by introducing a bias in the fiber orientation and found that the cell elongates in the direction of this bias and that FAs preferentially form at the poles of the cell. The model explains cell alignment to anisotropic collagen fiber networks (Lai et al., 2012; Chaubaroux et al., 2015; Sapudom et al., 2023). The model suggests that mechanosensitivity of the FAs is sufficient for the cell to sense the orientation of the fibers. This suggests that the stiffness-dependent maturation of FAs allows the cell to sense the orientation of the network. Others have suggested that a positive feedback loop between cell contractility and ECM stress drives cell elongation on anisotropic substrates, claiming that cells increase their contractile forces when sensing higher ECM stress (Alisafaei et al., 2022). This interesting explanation could be studied in further models. A conflicting observation, however, is that cells do not increase their contractile forces purely based on the stiffness of their environment (Feld et al., 2020).

After discussing the effect of pre-aligned ECM fibers on cell morphology, we studied the role of fiber orientation in mechanical cell–ECM reciprocity. Recently, it has been proposed that fiber reorientation by the cell is a two-way process: (1) cell protrusions adhere to and reorient fibers and (2) these fibers then develop anisotropic tension, which stabilizes the protrusions on aligned fibers, creating a self-reinforcing cycle (Alisafaei et al., 2022). Our model predicts a similar mechanism based on basic principles of mechanosensitive FA maturation. This mechanical reciprocity plays a role at larger multicellular scales. For example, in metastasis, the contractile forces of tumors align surrounding fibers (Balcioglu et al., 2016), and cancer cell migration is enhanced on aligned fibers (Sander, 2014; Doyle et al., 2022).

Our hybrid model predicts cell spreading, alignment, and ECM remodeling in terms of simple principles. However, we still could study to what extent it quantitatively matches experimental observations. One limitation to the model is its restriction to two dimensions. In 2D cell cultures, for example, cells extend protrusions beneath collagen fibers, wrapping around them (Friedrichs et al., 2007), a process not captured in two dimensions. Extensions capturing 3D fiber effects would require the use of a 3D or multi-layer CPM combined with additional cell-fiber behavior such as fiber repulsion. A second limitation to the ECM model is the lack of validation of its mechanical properties and network topology against real ECM structures, despite its demonstration of viscoelastic behavior (Tsingos et al., 2023).

Future work could incorporate more realistic ECM topologies, as modeled in previous studies by Davoodi Kermani et al. (2021) and Eichinger et al. (2021), which would significantly enhance the accuracy of this model. Possible other extensions include the study of cell migration along fibers by using one of the many active cell migration models implemented for the CPM such as a polarity vector (Beltman et al., 2007; Burger et al., 2022) or the Act model (Niculescu et al., 2015). An additional mechanism for FA breakdown is also needed because the current lifetimes of the FAs are unrealistically high and can even span nearly the whole simulated time (Supplementary Figure S9). The difficulty lies in the detachment of FAs at the rear of the migrating cell, which could be done, for example, by applying a model for asymmetric traction forces that would rupture the rear FAs or by introducing a chemical symmetry-breaking component (Yamaguchi and Knaut, 2022). To model cell migration in 3D, fiber exclusion should be added. In the current model, the cell interacts with the fibers only at adhesion sites, so the cell membrane could move through the fibers, which is not realistic. Another example of possible further study is that of multicellular mechanical interaction, which has been studied using CPM models (van Oers et al., 2014; Rens and Merks, 2017; Chiang and Chung, 2024). These studies applied a linear elastic continuous approach for modeling the ECM, whereas the realistic ECM has non-linear behavior such as strain stiffening. Such non-linear behavior is easily incorporated into the fibrous ECM model (Tsingos et al., 2023) and, when linked to our model of dynamic mechanosensitive FAs, could be used in modeling cell–cell mechanical communication. The present model could also be extended with the effect of proteolytic enzymes, such as MMPs, that would digest matrix fibers or cross-linkers, e.g., during angiogenesis, tissue remodeling, and tissue repair.

We demonstrated how the CPM can be used to study the mechanical reciprocity between a cell and a fibrous ECM, revealing that mechanosensitive adhesion explains the cell’s tendency to align with the dominant fiber orientation in the ECM, and their ability to reorient fibers to stabilize protrusions. This model can now be applied to systems in which these mechanisms are believed to play a key role.

## Data Availability

The raw data supporting the conclusions of this article will be made available by the authors, without undue reservation.
