# Supplementary material for: How cells align to structured collagen fibrils: a hybrid cellular Potts and molecular dynamics model with dynamic mechanosensitive focal adhesions
Source: Front Cell Dev Biol. 2025 Jan 6;12:1462277. doi: 10.3389/fcell.2024.1462277 (PMC11743931; doi:10.3389/fcell.2024.1462277)
Supplement: Supplementary file 4 [file DataSheet1.pdf]

Supplementary Material

1 SUPPLEMENTARY METHODS

1.1 Eccentricity and cell shape

To study cell elongation we fit an ellipse to the cell shape. We fit the major axis A and minor axis B of an ellipse such that its moment of inertia is equal to the moment of inertia of the cell shape. The details of this procedure can be found in the supplements of (Rens and Merks, 2017) . The eccentricity of the cell is then defined as

$$e = \sqrt{1 - \frac{B^2}{A^2}}. \tag{S1}$$

Clearly, $e \approx 1$ if the cell is elongated since in this case $A \gg B$. Likewise, $e = 0$ if $A = B$ which is the case when the cell is completely circular.

When the ellipse is fitted, we not only compute the length of the major axis and the minor axis, but also the unit vectors v_A and v_B such that the ellipse is parameterized by

$$c_{\text{cell}} + Av_a \sin(2\pi t) + Bv_B \cos(2\pi t) \tag{S2}$$

for $t \in [0, 1)$ and with c_{cell} the center of the cell.

The vector v_A is used to quantify the direction of the cell elongation. To be more precise, given a unit vector v in a direction to which we want to compare the direction of elongation, then the amount of alignment between v and v_A is the absolute value of the inner product between v and v_A. We take the absolute value since we do not want to distinguish the directions v from $-v$ nor the directions v_A from $-v_A$.

We quantified the difference in cell area, the number of lattice sites equal to the spin of the cell, from the starting area of the cell which is chosen to be 800 lattice sites.

1.2 Order Parameter

Measuring the alignment of directed objects arises repeatedly in mathematical modeling. The order parameter is a quantity that is 0 when these objects point in different directions, and is 1 if they point in the same direction. Here we used an order parameter to quantify the alignment of collagen strands. Specifically, we are interested in quantifying the alignment of a set of unit vectors $v_1, \ldots, v_m$ in the way of an order parameter.

A commonly used order parameter is obtained by measuring the angles of these unit vectors with the x-axis to obtain angles $\theta_1, \ldots, \theta_m \in [0, \pi)$. One has to be careful when measuring these angles as to obtain angles between 0 and π radians, always choosing the minimum of the angle between v_i and either $(1, 0)$ or $(-1, 0)$. Finally, the order parameter S_{angle} is the modulus of the complex number $\frac{1}{m}\sum_{j=1}^{m} \exp(2\theta_j i)$ with $i^2 = -1$.

We used a different but equivalent method to compute the order parameter which was inspired by the introduction of a paper on the theory of Q-tensors of liquid crystals (Borthagaray et al., 2020). This method does not require the careful angle measurements as it uses the vectors $v_1, \ldots v_m$ directly. We compute the

order parameter S as the largest eigenvalue of the Q-matrix

$$Q = \frac{1}{m}\sum_{j=1}^{m} 2v_i v_i^\top - I, \tag{S3}$$

with I the 2×2 identity matrix.

We will now show that these two methods are equivalent by showing that $S = S_{\text{angle}}$. First we measure the angles $\theta_1, \ldots, \theta_m$ as described above. Next, we consider the vectors $w_i = (\cos(\theta_i), \sin(\theta_i))$ for $i = 1, \ldots, m$. Since $w_i = \pm v_i$ and hence $w_i w_i^\top = v_i v_i^\top$ we compute that Q is of the form

$$Q = \frac{1}{m}\begin{pmatrix} \sum_{j=1}^{m}\cos(2\theta_j) & \sum_{j=1}^{m}\sin(2\theta_j) \\ \sum_{j=1}^{m}\sin(2\theta_j) & -\sum_{j=1}^{m}\cos(2\theta_j) \end{pmatrix}, \tag{S4}$$

where we used the standard trigonometric identities $2\cos^2(x) - 1 = \cos(2x) = 1 - 2\sin^2(x)$. The positive eigenvalue S of Q is then easily recognized, since Q is a symmetric traceless 2×2 matrix, as

$$S = \sqrt{\left(\frac{1}{m}\sum_{j=1}^{m}\cos(2\theta_j)\right)^2 + \left(\frac{1}{m}\sum_{j=1}^{m}\sin(2\theta_j)\right)^2},$$

which is equal to S_{angle}.

Now we use S in two different ways. To compute the global order parameter S_{global} and to compute the local order parameter S_{local}. The difference between S_{global} and S_{local} stems from the sampling of the unit vectors used to compute them.

The global order parameter S_{global} is computed as follows. Suppose that $x_1, \ldots, x_n \in \mathbf{R}^2$ are the positions of the beads of the network. Then for each bond (i, j) in the network we define $v_{(i,j)} = \frac{x_i - x_j}{\|x_i - x_j\|}$ and we compute the order parameter using all these vectors as input.

The local order parameter S_{local} is computed by binning the network in bins of size $l \times l$. Then, for each bin we compute the local order parameter with all bonds that fall in that bin as with the global order parameter. Some bonds are quite long and might cross a bin but might not give a contribution to the order parameter as both end points might lie outside of the bin. To remedy this, we refined the network by interpolating each bond (i, j) in the network with segments of length 1 before computing the local order parameter.

1.3 Measurement of time between onsets

We study locally the the rearrangement of collagen fibers and the spreading of the cell. As the CPM is a discrete model, there is no obvious continuous spreading parameter. We used the binning procedure as in the computation of the local order parameter. We binned the network into square bins of 5×5 lattice sites. For each of these lattice sites we computed the order parameter $S_{\text{local}}(t)$ over time and we quantified the amount of spreading in that bin as

$$C_{\text{local}}(t) = \frac{\text{number of positive spins in this bin}}{25}. \tag{S5}$$

This gives curves $S_{\text{local}}(t)$ and $C_{\text{local}}(t)$ as shown in Figure 4(A) in the main text.

Since we observe that these curves, when the cell makes a pseudopodium, both increase from low to high, we want to quantify the difference in onset of this increase. To this end, we fit sigmoidal functions

$$\sigma_{L,k,x_0}(t) = \frac{L}{1+\exp(-k(x-x_0))}, \tag{S6}$$

to the curves $S_{\text{local}}(t)$ and $C_{\text{local}}(t)$ and we obtain parameter sets $\left(L_{\text{cell}}, k_{\text{cell}}, x_{0,\text{cell}}\right)$ and $\left(L_{\text{S}}, k_{\text{S}}, x_{0,\text{S}}\right)$. The difference in onset between $S_{\text{local}}(t)$ and $C_{\text{local}}(t)$ is then the number $x_{0,S} - x_{0,\text{cell}}$.

2 SUPPLEMENTARY TABLES AND FIGURES

2.1 Figures

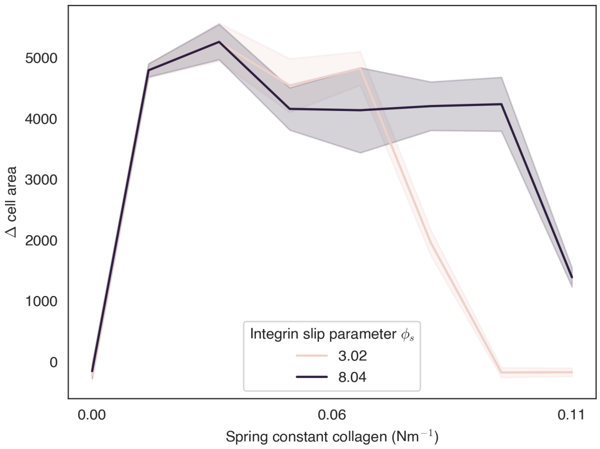

Figure S1. Final cell areas after spreading on a regular ECM with a contraction of 1. The ϕ_s parameter describes the slip-regime of the FAs. The cell spreads on stiffer substrates when a higher slip parameter is used.

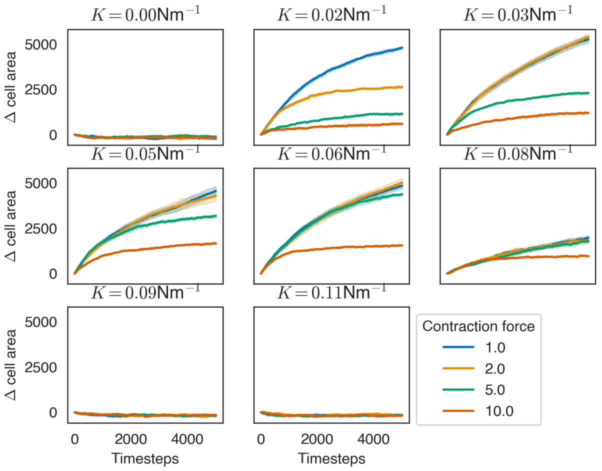

Figure S2. We investigated cell spreading on an isotropic matrix with varying stiffness and with varying cell contraction forces. On soft matrices, we see a large difference between contractile and non-contractile cells with contractile cells spreading the least. This difference decreases when the substrate stiffness is increased and finally disappears when the substrate is too stiff for the cell to spread at all.

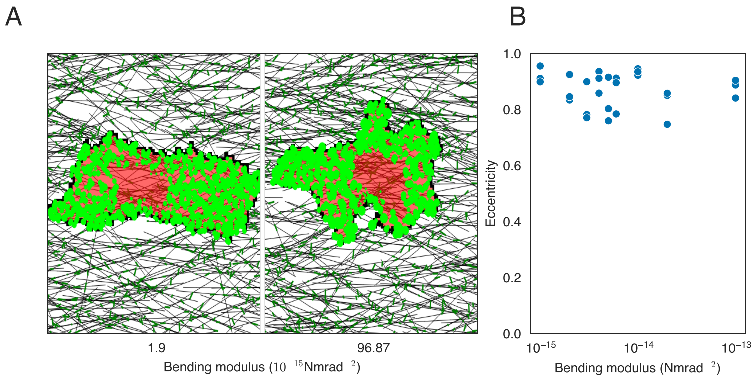

Figure S3. Here varied the bending modulus parameter of the fibers. With low bending modulus, we find the cell aligning with the fibers in the ECM. With higher bending modulus more protrusions orthogonal to the fiber orientation stabilize creating less smooth boundaries seeming to hinder cell alignment. However, the cellular eccentricities do not decrease on anisotropic ECMs with higher bending modulus. A: Screenshots of lower and higher bending modulus simulation. B: Eccentricities for different bending moduli.

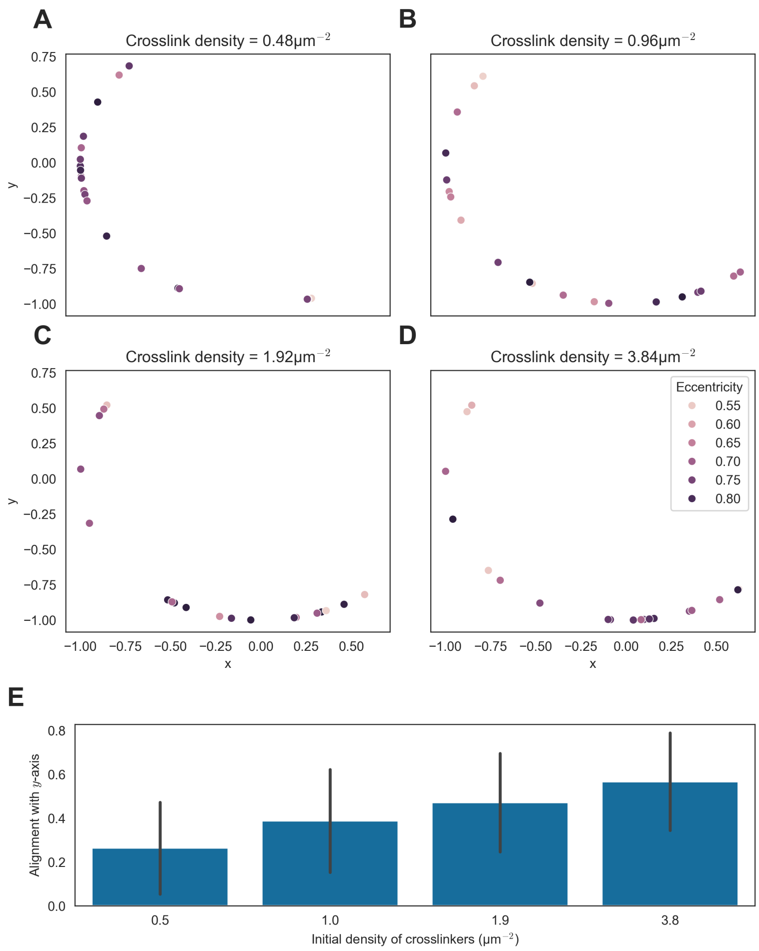

Figure S4. The cell elongates orthogonal to the substrate's orientation (the x-axis) when the substrate is stiff, here the spring constant is taken to be $0.031\,\mathrm{N m^{-1}}$. A-D: The major axis of an ellipse fitted to the cell is shown for different number of cross-linkers. For elongated cells, shown with a darker color, on highly cross-linked matrices, the major axis is close to the y-axis, showing that the cells are aligned to the y-axis. E: A barplot showing the degree of alignment with the y-axis for different cross-link densities.

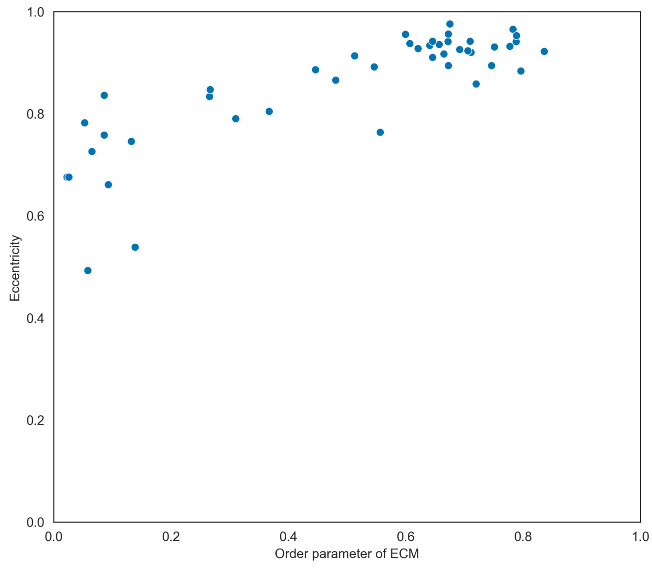

Figure S5. The relation of the global order parameter of the ECM on the final eccentricity of the cell. We see a gradual increasing effect, The relation between the cell's eccentricity and the order parameter of an ECM with a cross-link density of $3.8\,\mu\mathrm{m}^{-2}$ and fibers of stiffness $0.031\,\mathrm{Nm}^{-1}$.

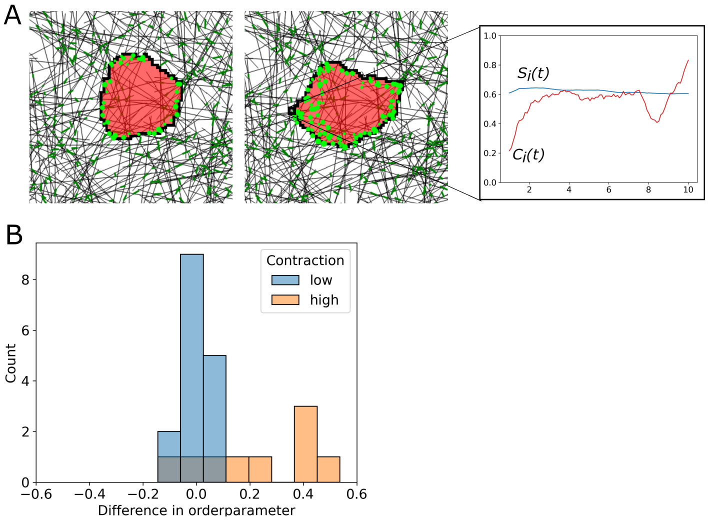

Figure S6. To illustrate that the cell does not remodel the ECM when contraction force is lowered to a tenth of the original force, we selected a protrusion on the right side of the cell and computed the functions $S_i(t)$ and $C_i(t)$ for 4×4 bins around the protrusion. The functions $S_i(t)$ and $C_i(t)$ do not show the same as in Figure 4(A) (A), and hence sigmoidial functions can not be fitted. As the contraction force is lowered, there is less remodeling of the network and S is close to constant while the cell spreads. Note that S is relatively high, hence the cell is able to spread here. We compared the difference in final remodeling between the high contraction force situation of Figure 4 and the lower contraction force by studying $\Delta S_i = S_i(10000) - S_i(0)$. We find a significance difference between the distributions, with higher contraction force leading to more realignment and lower contraction force leading to near zero alignment (B). A: Screen shot of initial configuration and final configuration and a pair of representative functions $S_i(t)$ and $C_i(t)$. B: Distribution of ΔS around a protrusion for a highly contractile cell and a less contractile cell. Means of the distributions are significantly different (Welch t-test, $p = 0.01$).

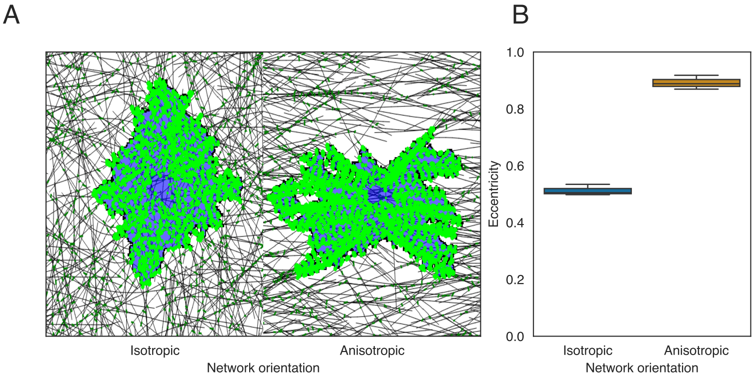

Figure S7. The CPM runs on a grid of fixed size. Here we double the CPM resolution to see if the cell alignment effects depends on grid size. The resolution is doubled by increasing the integration domain to 400×400, re-scaling the CPM-parameters according to Magno et al. (2015), and doubling the length of the strands. A: Screenshots of the high resolution simulation. B: Boxplots of eccentricities, showing that the cell alignment is not dependent on CPM resolution.

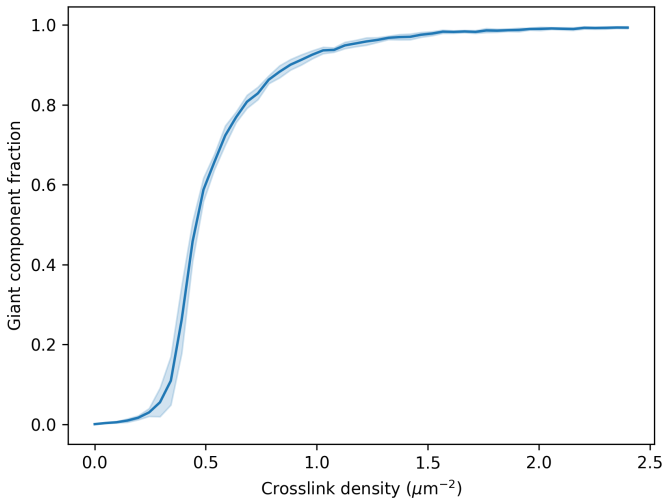

Figure S8. The percolation of the network was measured by computing the giant component, the number of beads of the largest connected component, for networks of different cross-linker densities. We repeated this computation 10 times for each network and we found a sharp transition in the giant component. We conclude that the percolation threshold of the type of networks discussed in this paper is around a cross-linking density of $1.0\mu\mathrm{m}^{-2}$.
.

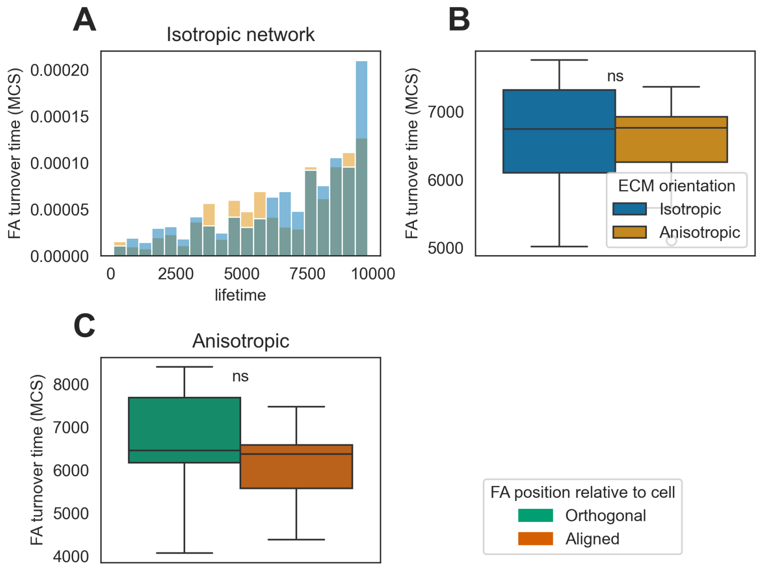

Figure S9. We studied average FA turnover times in isotropic and anisotropic ECMs. FA degradation is a complex process which involves different pathways that also effect cell polarity. In our model, FA degradation happens only at the cell-medium interface when the cell ruptures a FA. Hence, if FAs are transported away from the boundary their turnover time can be infinite. In this figure infinite turnover times are removed and hence only the FA turnover at the boundary is considered. We find no significant effects in FA turnover time between isotropic and anisotropic ECMs (A,B). FA orientation relative to the ECM anisotropy axis does not change FA turnover time (C).

Video S1: Cell on a regular, soft ECM does not spread fully ($K = 0.016\,\mathrm{Nm}^{-1}$).

Video S2: Cell on a regular, stiff ECM spreads to full size ($K = 0.062\,\mathrm{Nm}^{-1}$).

Video S3: Cell on a regular, stiff ECM spreads to full size ($K = 0.062\,\mathrm{Nm}^{-1}$, contraction $10\times$ higher).

Video S4: Cell spreading on a network-like ECM with soft fibers ($K = 0.031\,\mathrm{Nm}^{-1}$, cross-link density of $0.96, \mu\mathrm{m}^{-2}$).

Video S5: Cell spreading on a network-like ECM with stiff fibers ($K = 0.062\,\mathrm{Nm}^{-1}$), cross-link density of $0.96, \mu\mathrm{m}^{-2}$).

Video S6: Cell elongating on an anisotropic ECM with soft fibers ($K = 0.031\,\mathrm{Nm}^{-1}$, cross-link density of $0.96, \mu\mathrm{m}^{-2}$).

Video S7: Cell does not elongate on an anisotropic ECM with soft fibers ($K = 0.093\,\mathrm{Nm}^{-1}$, cross-link density of $0.96, \mu\mathrm{m}^{-2}$).

Video S8: Cell on anisotropic matrix with lower number of cross-linking ($K = 0.062\,\mathrm{Nm}^{-1}$, cross-link density of $0.48, \mu\mathrm{m}^{-2}$).

Video S9: Cell on anisotropic matrix with high cross-linking ($K = 0.062\,\mathrm{Nm}^{-1}$, cross-link density of $4.8, \mu\mathrm{m}^{-2}$).

Video S10: Cell on anisotropic matrix with high cross-linking and stiff fibers, elongating orthogonal to substrate orientation ($K = 0.078\,\mathrm{Nm}^{-1}$, cross-link density of $4.8, \mu\mathrm{m}^{-2}$).

REFERENCES

Borthagaray, J. P., Nochetto, R. H., and Walker, S. W. (2020). A structure-preserving FEM for the uniaxially constrained Q-tensor model of nematic liquid crystals. *Numerische Mathematik* 145, 837–881. doi:10.1007/s00211-020-01133-z

Magno, R., Grieneisen, V. A., and Marée, A. F. (2015). The biophysical nature of cells: Potential cell behaviours revealed by analytical and computational studies of cell surface mechanics. *BMC Biophysics* 8, 8. doi:10.1186/s13628-015-0022-x

Rens, E. G. and Merks, R. M. H. (2017). Cell Contractility Facilitates Alignment of Cells and Tissues to Static Uniaxial Stretch. *Biophysical Journal* 112, 755–766. doi:10.1016/j.bpj.2016.12.012
